# Auxin-to-Gibberellin Ratio as a Signal for Light Intensity and Quality in Regulating Soybean Growth and Matter Partitioning

**DOI:** 10.3389/fpls.2018.00056

**Published:** 2018-01-30

**Authors:** Feng Yang, Yuanfang Fan, Xiaoling Wu, Yajiao Cheng, Qinlin Liu, Lingyang Feng, Junxu Chen, Zhonglin Wang, Xiaochun Wang, Taiwen Yong, Weiguo Liu, Jiang Liu, Junbo Du, Kai Shu, Wenyu Yang

**Affiliations:** ^1^College of Agronomy, Sichuan Agricultural University, Chengdu, China; ^2^Sichuan Engineering Research Center for Crop Strip Intercropping System, Chengdu, China; ^3^Key Laboratory of Crop Ecophysiology and Farming System in Southwest, Ministry of Agriculture, Chengdu, China

**Keywords:** crop, growth, hormones, intercropping, light, morphology, shading

## Abstract

The intensity and quality (red to far-red (R/Fr) ratio) of light directly affect growth of plant under shading. Gibberellins (GAs) and auxin [indole-3-acetic acid (IAA)] play important roles in mediating the shading adaptive responses of plants. Thus, the intensity and quality of the uncoupling light from shading were assessed to identify the influence of each component on the morphology and matter distribution of the leaf, stem, and petiole. This assessment was based on the changes in endogenous Gibberellin 1 (GA1) and IAA levels. Soybean plants were grown in a growth chamber with four treatments [normal (N), N+Fr, low (L), and L+Fr light]. Results revealed that the reductions in photosynthetically active radiation (PAR) and R/Fr ratio equally increased height and stem mass fractions (SMFs) of the soybean seedling. The light intensity significantly influenced the dry mass per unit area and mass fraction of soybean leaves, whereas the light quality regulated the petiole elongation and mass fraction. Low R/Fr ratio (high Fr light) increased the soybean biomass by improving the photosynthetic assimilation rate and quantum yield of photosystem II. In addition, the IAA and GA1 levels in the leaf, stem, and petiole did not reflect the growth response trends of each tissue toward light intensity and quality; however, trends of the IAA-to-GA1 content ratios were similar to those of the growth and matter allocation of each soybean tissue under different light environments. Therefore, the response of growth and matter allocation of soybean to light intensity and quality may be regulated by the IAA-to-GA1 content ratio in the tissues of the soybean plant.

## Introduction

Light is one of the most important environmental factors because it regulates photosynthetic assimilation and partitioning in plants (Slewinski and Braun, 2010; Jiang et al., 2011). Plants use several types of photoreceptors, such as phytochromes and cryptochromes, to perceive aspects of radiation in the environment (Possart et al., 2014; Ballaré and Pierik, 2017; Mawphlang and Kharshiing, 2017). However, shading reduces the intensity and changes the spectral composition of the light (Yang et al., 2014). For example, reductions in the red-to-far-red (R/Fr) light ratio and the photosynthetically active radiation (PAR) strongly impair the growth and development of plants under shading (Kurepin et al., 2007a; Yang and Li, 2017).

Plants can adjust their morphology and physiology to acclimatize toward a modified light quality (e.g., reduced R/Fr ratio) and a decreased PAR (Kurepin et al., 2007a; Valladares and Niinemets, 2008). This acclimatization results in the allocation of carbon to the elongation of stem and petiole at the expense of leaf and root development (Gommers et al., 2013; Park and Runkle, 2017). Previous studies on plant shading responses analyzed the morphological plasticity and matter allocation pattern of the whole plant (Li et al., 2010; Marchiori et al., 2014; Rodríguez-López et al., 2014; Wu et al., 2017b); however, the light environment treatments were set through artificial shading with shade nets or screens, but do not change the spectral composition (Li et al., 2014). Although many studies investigated the effects of the interaction between the quality (R/Fr ratio) and intensity of light on the growth of hypocotyls (Kurepin et al., 2007a) or anatomical structure of leaves (Wherley et al., 2005), these studies focused only on the responses of single organs and not of the whole plant.

Light-dependent changes in plant morphogenesis are regulated by plant hormones (Ashraful Islam et al., 2014; Kissoudis et al., 2017; Wu et al., 2017a). Among the endogenous plant hormones, gibberellins (GAs) and auxin [indole-3-acetic acid (IAA)] mediate the shading adaptive responses of plants, especially for shade-induced differential growth and elongation (Kurepin et al., 2007a; de Lucas and Prat, 2014; Yang and Li, 2017). Low values of R/Fr ratio and PAR promoted growth and increased the GA levels in the internodes of bean plants (Beall et al., 1996), in the hypocotyls and leaves of sunflower (Kurepin et al., 2007b), and in the shoots of tomato (Kurepin et al., 2006) and Arabidopsis (Kurepin et al., 2012). A low R/Fr ratio coupled with a normal PAR increased the endogenous IAA levels in the third internode of *Pisum sativum* seedlings (Behringer and Davies, 1992) and in the leaves of sunflower (Kurepin et al., 2007b). Although shading promotes the elongation of petioles, the mechanism on how the intensity and quality of light affect the GA and IAA levels of petioles needs further study (Morelli and Ruberti, 2000). Therefore, the relationship between the growth of each plant organ and the associated hormones (GAs and IAA) must be further investigated to reveal the morphological response of plants to the interaction between light quality and intensity. A few studies reported that the specific distributions of GAs and IAA in radish are strongly correlated with the photo-morphogenetic responses to blue or red light (Kara et al., 1997).

Soybean [*Glycine max* (L.) Merr.] is the fourth most widely cultivated crop worldwide, and its main products include protein and oil (Tacarindua et al., 2013; Wu et al., 2017a). Planting density and planting pattern are the two key factors in increasing soybean yield, particularly in close planting and intercropping (Yang et al., 2014). However, soybean plants suffer from mutual shading when planted closely or intercropped with tall, neighboring vegetation (Yang et al., 2014; Zhang et al., 2014). Change trends were found in the height, morphological characteristics, and matter distribution of soybean plant under shade conditions (Yang et al., 2014; Wu et al., 2017b). Thus, the light intensity and quality must be investigated separately from shading to determine how soybean responds to a low PAR coupled with normal R/Fr ratio and to a low R/Fr ratio coupled with normal PAR. In addition, the factors mediating these responses must be analyzed.

The objectives of this study were as follows: (i) to analyze the effects of the interaction between the intensity and quality of light on the morphology and matter distribution in the leaf, stem, and petiole; (ii) to examine how light intensity and quality affect matter assimilation based on photosynthesis and chlorophyll (Chl) fluorescence analysis; and (iii) to identify the roles of each light component in the morphology and matter distribution of the leaf, stem, and petiole based on the changes in endogenous GA1 and IAA levels.

## Materials and Methods

### Plant Material and Growing Conditions

Soybean seeds (Nandou 12, Nanchong Academy of Agricultural Science, Sichuan, China) were soaked in wet filter paper for 1 day at 30°C. The germinated seeds were planted in containers (40 cm in length, 20 cm in width, and 15 cm in height) filled with humidified organic soil with a seedling spacing of 10 cm. The plants were grown in growth chambers. The temperature was maintained at 25°C during 12 h of daytime and at 20°C during 12 h of nighttime. The relative humidity was approximately 60%. The soybean seedlings were watered every 2 days with 0.2% Hoagland’s solution (Hoagland and Arnon, 1950). Upon the development of the first trifoliolate leaf (before the second trifoliolate leaf appeared), the seedlings were then divided into four groups for different light environment treatments. After 15 days of treatment, the plants were sampled to measure their morphological and physiological parameters.

**FIGURE 1:**
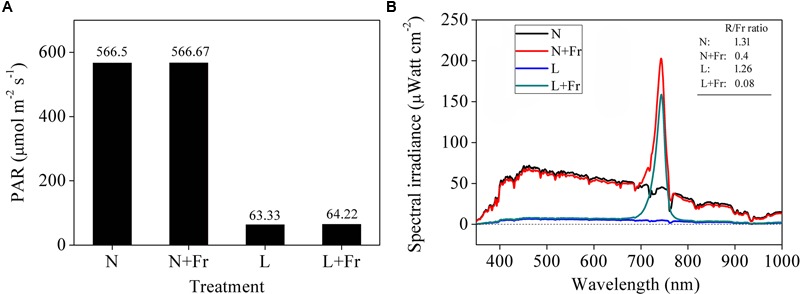
Light intensity **(A)** and quality **(B)** of soybean canopy under different treatments. N, N+Fr, L, and L+Fr denote normal light, normal light plus far-red light, low light, and low light plus far-red light, respectively.

Combinations of black nylon net and far-red light-emitting diode (LED) (36 W, light peaking at 735 nm) light sources were used to adjust the light intensity (PAR) and quality (R/Fr ratio) in the growth chambers. The soybean seedling canopy at 50 cm height was covered with a piece of black nylon net to reduce the PAR and was added with two far-red LED modulator tubes to adjust the R/Fr ratio. The following four treatments were used (**Figure 1**): normal light (N; PAR: 566.50 μmol m^-2^s^-1^; R/Fr ratio: 1.30), normal light plus far-red light (N+Fr; PAR: 566.67 μmol m^-2^s^-1^; R/Fr ratio: 0.40), low light (L; PAR: 63.33 μmol m^-2^s^-1^; R/Fr ratio: 1.26), and low light plus far-red light (L+Fr; PAR: 64.22 μmol m^-2^s^-1^; R/Fr ratio: 0.08). The PAR was measured by using LI-190SA quantum sensors (LI-COR Inc., Lincoln, NE, United States) placed at 10 cm above the soybean canopy. The spectral irradiance of the different wavelengths in the soybean canopy was measured using a fiber-optic spectrometer (AvaSpec-2048; Avantes, Netherlands) (Yang et al., 2014). The sensor has a field view of 25°, and a full sky irradiance remote cosine corrector. The spectral irradiance was originally measured at wavelengths ranging from 400 to 1000 nm at 0.6 nm intervals.

### Measurement of Morphological Characteristics

The height from the soil surface and the petiole length of the second trifoliolate leaves of five soybean seedlings were measured 2 weeks after the plants were subjected to the four treatments. The leaf dry mass per unit area (*M*_A_) was calculated by using the proportion of the second trifoliolate leave biomass in the total leaf area. The plants were harvested and separated into leaves, stems, and petioles. Samples were over-dried at 105°C for 0.5 h to destroy the tissues and then dried at 80°C for 72 h to a constant weight. Afterward, the dry weights of the leaves, stems, and petioles were measured. The total biomass, leaf mass fraction (LMF), SMF, and petiole mass fraction (PMF) were then obtained based on the previous data (Rodríguez-López et al., 2014).

### Determination of Photosynthesis and Photochemistry

The second trifoliolate leaves of five plants under each treatment were used to analyze the photosynthetic and photochemical responses to the changes in light intensity and quality at around 10:00 AM. Leaf gas exchange analysis was evaluated in a 6 cm^2^ leaf chamber with a CO_2_ concentration of 400 μ mol mol^-1^ by using a portable infrared gas analyzer (Li-6400, LI-COR Inc., Lincoln NE, United States). Eleven light intensity levels (0, 20, 50, 100, 150, 200, 400, 600, 800, 1000, and 1200 μmol m^-2^s^-1^ at a time step of 2 min) were imposed. The photosynthetic response curve to light under different treatments was fitted with a previously reported equation (Marchiori et al., 2014). The maximum photosynthetic rate (*P*_max_), light compensation point (LCP), light saturation point (LSP), and apparent quantum yield (AQY) were then estimated using the method proposed by Gong et al. (2015).

The leaves under the four treatments were simultaneously darkened for 10 min prior to measurement. Images of the minimum Chl fluorescence yield (*F*_o_) in the dark-adapted state were captured using low-frequency light pulses (1 Hz). The maximum fluorescence (*F*_m_) was determined by applying a blue saturation pulse (10 Hz). The maximum quantum yield of the photosystem II (PSII) photochemistry (*F*_v_/*F*_m_ ratio) was determined as *F*_m_ -*F*_o_/*F*_m_, and images were captured. Actinic illumination (750 μmol m^-2^ s^-1^) was switched on, and saturating pulses were applied at 20 s intervals for 15 min to determine the *F*_m_ and Chl fluorescence during actinic illumination (*F*_s_). The quantum efficiency of the PSII photochemistry and the non-photochemical quenching (NPQ) were calculated according to the method by Calatayud et al. (2006).

### Analysis of GAs and IAA

The leaf and petiole of the second trifoliolate leaf and the top internode per soybean plant under different treatments were collected. Three replicates were measured for each treatment. Each replicate included four plants. In this study, endogenous GA1 was analyzed according to previous studies. The bioactive GAs are GA1, GA3, GA4, and GA7 (Yamaguchi, 2008). The presence of GA1 in higher plant species suggests that it is a common bioactive GA (MacMillan, 2001; Li et al., 2016; Sheerin and Hiltbrunner, 2017). The samples were weighted, immediately frozen in liquid nitrogen, and then stored at -80°C to measure the endogenous hormones and analyze the differential proteins.

Approximately 5 mg of the samples were frozen in liquid nitrogen and finely ground. Extraction with 80% methanol (MeOH) (methanol/H_2_O, 80/20, v/v) was then performed at 4°C for 12 h. [^2^H_5_] IAA (15.0 ng g^-1^), [^2^H_2_] GA1 and GA4 (1.00 ng g^-1^) were added to the plant samples as internal standards prior to grinding. After purifying with a C_18_ preparative column (C_18_-PC), the 80% MeOH eluate was evaporated under mild nitrogen stream at 35°C, redissolving in 100 μL H_2_O, and injected into the nano-liquid chromatography-electrospray ionization-quadrupole-time of flight-mass spectrometry (nano-LC–ESI-QTOF-MS) system to identify and quantify the GA1, GA4, and IAA levels. All nano-LC experiments were conducted on a Shimadzu Prominence nano-flow liquid chromatography system (Kyoto, Japan) with two LC-20AD nanopumps, two vacuum degassers, a LC-20AB HPLC pump, a SIL-20AC HT autosampler and a FCV nano valve. MS analysis was performed using a microTOF-Q orthogonal-accelerated TOF mass spectrometer (Bruker Daltonics, Bremen, Germany) controlled by Bruker Daltonics Control 3.2. Data were analyzed using the Bruker Daltonics data analysis 3.4 software. Details on the analysis of IAA, GA1, and GA4 levels can be found at Chen et al. (2012).

### Analysis of Differentially Expressed Proteins

Tandem mass tag (TMT) technique was performed under different intensity and quality conditions to examine the differentially expressed proteins associated with auxin and GAs that regulate stems and petioles. Three replicates were measured for each treatment.

#### Protein Extraction

The sample was ground using liquid nitrogen into cell powder and then transferred to a 5 mL centrifuge tube. Thereafter, four volumes of lysis buffer [8 M urea, 1% Triton-100, 10 mM dithiothreitol (DTT), and 1% protease inhibitor cocktail] were added to the cell powder, which was sonicated three times on ice using a high intensity ultrasonic processor (Scientz). The remaining debris was removed through centrifugation at 20,000 *g* at 4°C for 10 min. Finally, the protein was precipitated with cold 20% TCA for 2 h at -20°C. After centrifugation at 12,000 *g* at 4°C for 10 min, the supernatant was discarded. The remaining precipitate was washed with cold acetone three times. The protein was redissolved in 8 M urea and the protein concentration was determined using a BCA kit.

#### Trypsin Digestion

After removing the high abundance proteins, the serum was diluted with 8 M urea and reduced with 10 mM DTT for 1 h at 37°C. The sample was alkylated with 20 mM IAA for 45 min at room temperature in darkness. For trypsin digestion, the serum sample was diluted by adding 100 mM TEAB to the urea concentration of less than 2 M. Finally, trypsin was added at 1:50 trypsin-to-protein mass ratio for the first digestion overnight and 1:100 trypsin-to-protein mass ratio for the second 4 h-digestion. Approximately 50 μg of proteins for each sample were digested with trypsin for the following experiments.

#### TMT Labeling

After trypsin digestion, peptides were desalted using Strata X C18 SPE column (Phenomenex) and vacuum-dried. The peptides were reconstituted in 0.5 M TEAB and processed according to the manufacturer’s protocol for TMT 10-plex kit. Briefly, one unit of TMT reagent (defined as the amount of reagent required to label 100 μg of proteins) was thawed and reconstituted in 24 μL of ACN. The peptide mixture was then incubated for 2 h at room temperature, pooled, desalted, and dried by vacuum centrifugation.

#### HPLC Fractionation

The sample was then fractionated using high-pH reverse-phase HPLC using Agilent 300Extend C18 column (5 μm particles, 4.6 mm ID, 250 mm length). The peptides were first separated into 80 fractions with a gradient of 2–60% acetonitrile in 10 mM ammonium bicarbonate pH 10 over 80 min. The peptides were combined into 18 fractions and dried using vacuum centrifugation.

#### LC-MS/MS Analysis

The peptides were dissolved in 0.1% FA, and directly loaded onto a reversed-phase analytical column (Acclaim PepMap RSLC, Thermo Scientific). The gradient increased from 7 to 25% solvent B (0.1% FA in 98% ACN) in over 26 min, from 25 to 38% in 8 min, to 80% in 3 min, and then held at 80% for the last 3 min, all at a constant flow rate of 350 nL/min on an EASY-nLC 1000 UPLC system.

The peptides were subjected to NSI source and tandem mass spectrometry (MS/MS) in Orbitrap Fusion^TM^ Tribrid^TM^ (Thermo) coupled online with UPLC. Intact peptides were detected in the Orbitrap at a resolution of 60,000. The peptides were selected for MS/MS using NCE setting of 35, and ion fragments were detected in the Orbitrap at a resolution of 30,000. A data-dependent procedure that alternated between one MS scan and 10 MS/MS scans was applied for the top 10 precursor ions at a threshold intensity greater than 5E3 in the MS survey scan with 30.0 s dynamic exclusion. The electrospray voltage applied was 2.0 kV. Automatic gain control was used to prevent overfilling of the Orbitrap; 5E4 ions were accumulated for the generation of MS/MS spectra. For MS scans, the m/z scan range was from 350 to 1550. Fixed first mass was set at 100 m/z.

#### Database Search

The resulting MS/MS data were processed using MaxQuant with an integrated Andromeda search engine (v.1.5.2.8). Tandem mass spectra were searched against Uniprot *G. max* (*Linn.*) *Merr* database concatenated with reverse decoy database. Trypsin/P was specified as cleavage enzyme allowing up to two missing cleavages. Mass error was set to 10 ppm for precursor ions and 0.02 Da for fragment ions. Carbamidomethylation on Cys was specified as fixed modification, and oxidation on Met and acetylation on protein N-terminal were specified as variable modifications. False discovery rate thresholds for protein, peptides, and modification sites were specified at 1%. Minimum peptide length was set at 7. For the quantification method, TMT-10plex was selected. All the other parameters in MaxQuant were set to default values.

### Statistical Analysis

Data analysis was accomplished using the analysis of variance (ANOVA) on SPSS software (version 16.0). The differences among the four treatments were determined using Duncan’s multiple range test at the 0.05 level. The graphics program Origin Pro (version 8.0) was used for creating the illustrations.

## Results

### Morphological Parameters

**FIGURE 2:**
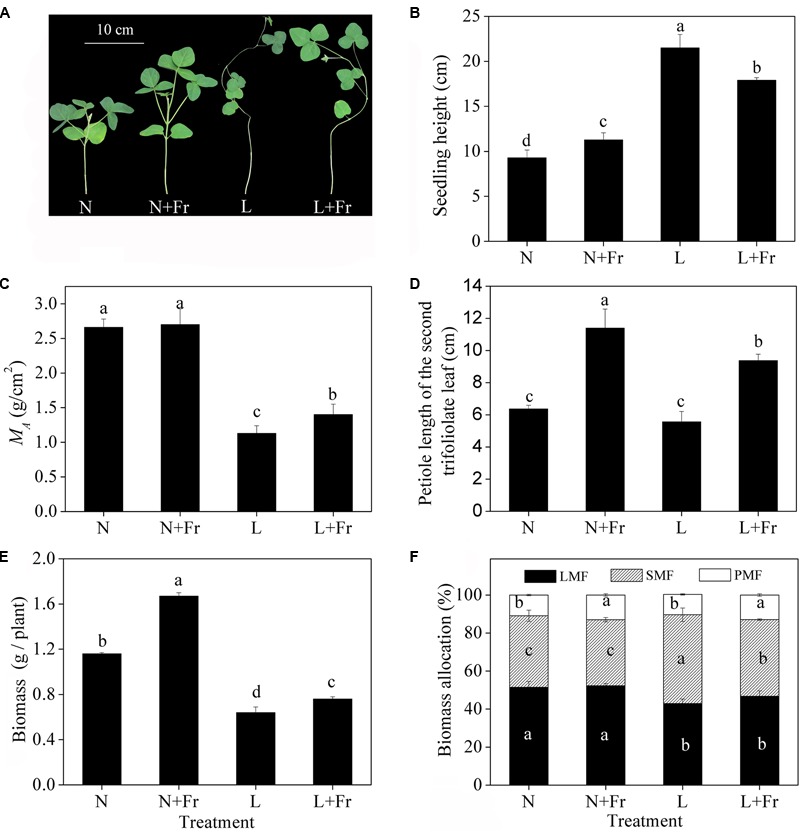
Seedling height **(B)**, leaf dry mass per unit area (*M*_A_) **(C)**, petiole length of the second trifoliolate leaf **(D)**, biomass **(E)**, and biomass allocation traits **(F)** (LMF, SMF, and PMF) of soybean under different light intensity and quality treatments. N, N+Fr, L, and L+Fr denote normal light (normal PAR and normal R/Fr ratio), normal light plus far-red light (normal PAR and low R/Fr ratio), low light (low PAR and normal R/Fr ratio), and low light plus far-red light (low PAR and low R/Fr ratio), respectively. LMF, SMF, and PMF represent leaf mass fraction, stem mass fraction, and petiole mass fraction, respectively. **(A)** Soybean seedlings grown for 35 days under different treatments. Each value is expressed as the mean ± SE. The means for each treatment that do not have a common letter are significantly different at *P* = 0.05, according to Duncan’s multiple range test.

**Figure 2** describes the morphological parameters of the soybean seedlings under different light intensity and quality treatments. Compared with that under N treatment, the seedling heights were significantly increased under N+Fr, L, and L+Fr treatments. The seedling heights were increased by 90.6 and 58.7%, whereas *M*_A_ was decreased by 58.1 and 48.1%, under L and L+Fr treatments, respectively, with respect to that under N+Fr treatment. No significant difference in *M*_A_ was observed between the N and N+Fr treatments. In addition, the petiole lengths under N and L treatments were significantly lower than those under N+Fr and L+Fr treatments. These results indicated that under the shade condition, the interaction between light intensity and quality (reduced R/Fr ratio) affected the seedling height of soybean, light intensity mainly affected *M*_A_, and light quality (R/Fr ratio) primarily influenced the petiole length.

Significant differences in the biomass were observed among the four treatments (**Figure 2E**). The biomass under N and N+Fr treatments was significantly higher than that under L and L+Fr treatments. However, the biomass of the soybean seedling under N+Fr and L+Fr treatments was increased by 43.2 and 18.1% with respect to those under N and L treatments, respectively, implying that the low R/Fr ratio (high far-red light) under N and L treatments increased the biomass of soybean. The biomass allocation traits are presented in **Figure 2F**. The LMFs under L and L+Fr treatments were significantly lower than those under N and N+Fr treatments. Opposite results were observed for SMF. In addition, PMF values of soybean were increased under N+Fr and L+Fr treatments with respect to the PMF under N and L treatments.

### Photosynthetic and Chl Fluorescence Characteristics

**FIGURE 3:**
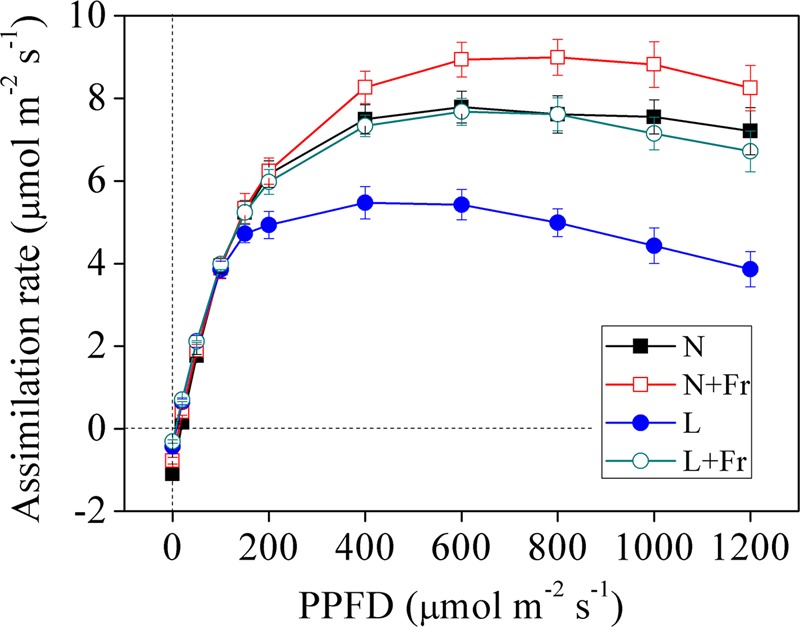
Light response curves of net assimilation rate under different light intensity and quality treatments. N, N+Fr, L, and L+Fr denote normal light (normal PAR and normal R/Fr ratio), normal light plus far-red light (normal PAR and low R/Fr ratio), low light (low PAR and normal R/Fr ratio), and low light plus far-red light (low PAR and low R/Fr ratio), respectively. PPFD represents the photosynthetic photon quanta flux density. Each value is expressed as the mean ± SE.

**Figure 3** shows the light response curves of the assimilation rate vs. the photosynthetic photon quanta flux density (PPFD) of the four treatments. The assimilation rates under N, N+Fr, L, and L+Fr treatments initially increased with increasing irradiation ranging from 0 to 200 μmol m^-2^s^-1^. The increasing trend subsequently plateaued and eventually reached a saturation point. The assimilation rates of the four treatments then gradually decreased as the PPFD increased. The assimilation rates of the four treatments presented no significant difference under low irradiance (<200 μmol m^-2^s^-1^). As the PPFD continued to increase, the light response curves of the net assimilation rate under N+Fr treatment became higher than that those of other treatments.

**Table 1** presents the analysis of the maximum photosynthetic rate (*P*_max_), LCP, LSP, and AQY, all of which were obtained based on the light response curves of the assimilation rates. The maximum values of *P*_max_ and LSP, which appeared under N+Fr treatment, were 9.01 μmol CO_2_ m^-2^s^-1^ and 863.64 μmol m^-2^s^-1^, respectively. *P*_max_ and LSP decreased by 14.87 and 47.37% under N treatment, 35.96 and 63.16% under L treatment, and 15.98 and 40.35% under L+Fr treatment with respect to the corresponding values under N+Fr treatment. In terms of the LCP value, the treatments can be ordered from highest to lowest as follows: N > N+Fr > L > L+Fr treatments. In addition, no significant differences were observed in AQY among the four treatments. These results implied that a reduced R/Fr ratio (increased far-red light) under normal or low light improved the photosynthetic and matter accumulation abilities of soybean seedlings.

**Table 1:** Light response model parameters of soybean seedlings under different light intensity and quality treatments.

Treatment	*P*_max_ (μmol CO_2_ m^-2^ s^-1^)	LCP (μmol m^-2^ s^-1^)	LSP (μmol m^-2^ s^-1^)	AQY (μmol μmol^-1^)
N	7.67 ± 0.2^b^	18.67 ± 2.12^a^	454.54 ± 22.54^c^	0.050 ± 0.007^a^
N+Fr	9.01 ± 0.17^a^	13.13 ± 1.58^b^	863.64 ± 30.31^a^	0.046 ± 0.005^a^
L	5.77 ± 0.31^c^	6.13 ± 2.07^c^	318.18 ± 7.58^d^	0.043 ± 0.003^a^
L+Fr	7.57 ± 0.35^b^	4.52 ± 1.05^c^	515.15 ± 22.73^b^	0.043 ± 0.003^a^

*N, N+Fr, L, and L+Fr denote normal light (normal PAR and normal R/Fr ratio), normal light plus far-red light (normal PAR and low R/Fr ratio), low light (low PAR and normal R/Fr ratio), and low light plus far-red light (low PAR and low R/Fr ratio), respectively. *P*_*max*_, LCP, LSP, and AQY represent the maximum photosynthetic rate, the light compensation point, the light saturation point, and the apparent quantum yield. Each value is expressed as the mean ± SE. The means for each treatment that do not have a common letter are significantly different at *P* = 0.05, according to Duncan’s multiple range test.*

**FIGURE 4:**
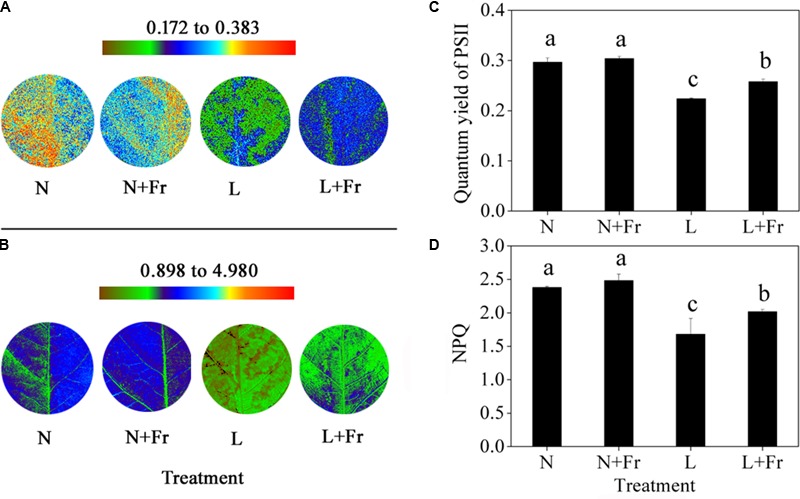
Quantum yield of PSII **(A,C)** and NPQ **(B,D)** of Chl fluorescence under different light intensity and quality treatments. Chl fluorescence images **(A,B)** were captured from a single plant in each treatment. The false color code depicted at the top of each image ranges from brown to red. N, N+Fr, L, and L+Fr denote normal light (normal PAR and normal R/Fr ratio), normal light plus far-red light (normal PAR and low R/Fr ratio), low light (low PAR and normal R/Fr ratio), and low light plus far-red light (low PAR and low R/Fr ratio), respectively. Each value is expressed as the mean ± SE. The means for each treatment that do not have a common letter are significantly different at *P* = 0.05, according to Duncan’s multiple range test.

The quantum yield of PSII is the fraction of light absorbed by leaves for photochemical electron transport (Maxwell and Johnson, 2000). The activation of NPQ dumps a significant fraction of excitation energy to partially avoid photoinhibition (Kanazawa and Kramer, 2002). In the present study, the quantum yields of PSII and NPQ under N and N+Fr treatments were significantly higher than those under L and L+Fr treatments, although no significant difference was found between the N and N+Fr treatments. Furthermore, a reduced R/Fr ratio increased the quantum yield of PSII and NPQ by 15.18 and 19.92% under L+Fr treatment, respectively, with respect to those under L treatment (**Figure 4**).

### Endogenous IAA, GA1, and GA4 Levels

**FIGURE 5:**
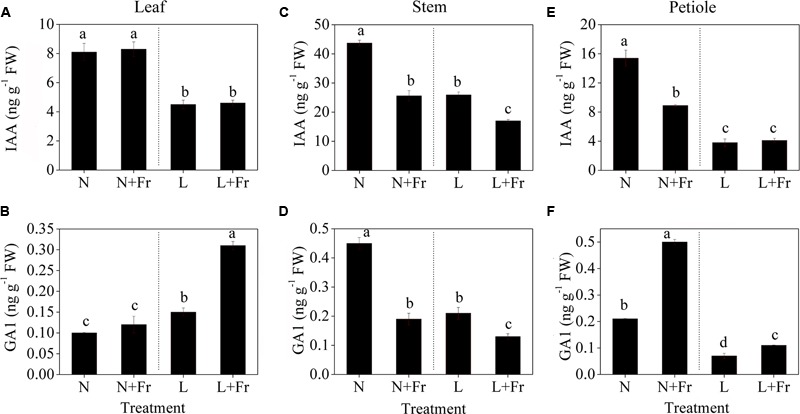
Auxin (IAA) and gibberellin 1 (GA1) levels in different tissues [leaf **(A,B)**, stem **(C,D)**, and petiole **(E,F)**] of soybean seedlings harvested after 15 days under different light intensity and quality treatments. The leaves and petioles were obtained from the second trifoliolate leaves, and the stem samples were collected from the top internodes. N, N+Fr, L, and L+Fr denote normal light (normal PAR and normal R/Fr ratio), normal light plus far-red light (normal PAR and low R/Fr ratio), low light (low PAR and normal R/Fr ratio), and low light plus far-red light (low PAR and low R/Fr ratio), respectively. Each value is expressed as the mean ± SE. The means for each treatment that do not have a common letter are significantly different at *P* = 0.05, according to Duncan’s multiple range test.

**Figure 5** shows the effects of light intensity and quality on the endogenous IAA, GA1, and GA4 levels in the leaf, stem, and petiole of soybean seedlings. Reduction of the PAR irradiance significantly decreased the endogenous IAA level of leaves but considerably increased the endogenous GA1 level. In comparison, reduction of the R/Fr ratio in presented no significant effect on the IAA level in the soybean leaves under N+Fr and L+Fr treatments with respect to that under N and L treatments, respectively (**Figure 5A**). Similar results were observed for the GA1 level between the N and N+Fr treatments. Nevertheless, low values of both R/Fr ratio PAR irradiance resulted in increased GA1 levels in the soybean leaves compared with that under normal R/Fr ratio coupled with low PAR irradiance (**Figure 5B**). In addition, the contents of GA4 in leaf, stem, and petiole were lower than GA1, and even GA4 contents of leaf and stem could not be detected under different treatments (**Table 2**). These results implied that light intensity played an important role in regulating the IAA and GA1 contents of leaves.

**Table 2:** Gibberellin 4 (GA4) levels in different tissues (leaf, stem, and petiole) of soybean seedlings harvested after 15 days under different light intensity and quality treatments.

Treatment	Leaf (ng g^-1^ FW^-1^)	Stem (ng g^-1^ FW^-1^)	Petiole (ng g^-1^ FW^-1^)
N	n.d.	0.15 ± 0.01^a^	0.06 ± 0.01^a^
N+Fr	n.d.	0.04 ± 0.00^b^	0.03 ± 0.00^b^
L	n.d.	n.d.	0.02 ± 0.00^c^
L+Fr	n.d.	n.d.	0.02 ± 0.01^bc^

*The leaves and petioles were obtained from the second trifoliolate leaves, and the stem samples were collected from the top internodes. N, N+Fr, L, and L+Fr denote normal light (normal PAR and normal R/Fr ratio), normal light plus far-red light (normal PAR and low R/Fr ratio), low light (low PAR and normal R/Fr ratio), and low light plus far-red light (low PAR and low R/Fr ratio), respectively. n.d. represents “not detected”. Each value is expressed as the mean ± SE. The means for each treatment that do not have a common letter are significantly different at *P* = 0.05, according to Duncan’s multiple range test.*

The IAA and GA1 levels in the top internodes of soybean stems exhibited the same change trends under different treatments (**Figures 5C,D**). A reduced R/Fr ratio or a low PAR irradiance decreased the IAA and GA1 levels in soybean stems with respect to the corresponding levels under N treatment. The IAA and GA1 levels under N+Fr treatment (reduced R/Fr ratio coupled with normal PAR irradiance) decreased by 41.42 and 57.78% from their corresponding values under N treatment. Similarly, the IAA and GA1 levels under L+Fr treatment (reduced R/Fr ratio coupled with low PAR irradiance) decreased by 34.36 and 38.10% from their corresponding values under L treatment (low PAR irradiance). These results implied that light intensity and quality jointly affected the IAA and GA1 levels, which regulated the stem growth.

The IAA levels of the soybean petioles under normal light conditions (N and N+Fr treatments) were significantly higher than those under low light conditions (L and L+Fr treatments) (**Figures 5E,F**). Nevertheless, a reduced R/Fr ratio under N+Fr treatment resulted in lower IAA levels in the petioles compared with that under N treatment. By contrast, the GA1 levels in petioles were significantly higher under N+Fr and L+Fr conditions (reduced R/Fr ratio) than those under N and L conditions (normal R/Fr ratio), respectively. Moreover, a low PAR irradiance decreased in the GA1 levels.

**FIGURE 6:**
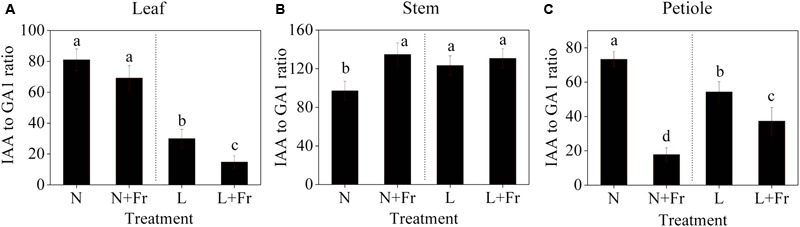
IAA-to-GA1 ratios in the different tissues [leaf **(A)**, stem **(B)**, and petiole **(C)**] of soybean seedlings harvested after 15 days under different light intensity and quality treatments. The leaves and petioles were obtained from the second trifoliolate leaves, and the stem samples were collected from the top internodes. N, N+Fr, L, and L+Fr denote normal light (normal PAR and normal R/Fr ratio), normal light plus far-red light (normal PAR and low R/Fr ratio), low light (low PAR and normal R/Fr ratio), and low light plus far-red light (low PAR and low R/Fr ratio), respectively. Each value is expressed as the mean ± SE. The means for each treatment that do not have a common letter are significantly different at *P* = 0.05, according to Duncan’s multiple range test.

**Figure 6** shows the IAA-to-GA1 ratios in each tissue of the soybean plant under different light intensity and quality treatments. A low PAR significantly decreased the IAA-to-GA1 ratio in the soybean leaves; however, no significant difference was observed in the IAA-to-GA1 ratios under N and N+Fr (reduced R/Fr ratio in normal light) treatments (**Figure 6A**). In the top internode of the stem, a low PAR or R/Fr ratio significantly increased the IAA-to-GA1 ratio (**Figure 6B**). Although the low PAR decreased the IAA-to-GA1 ratio of the soybean petioles, the reduced R/Fr ratio played a key role in regulating the petiole elongation under normal or low PAR conditions. For example, the IAA-to-GA1 ratios decreased by 75.7 and 31.4% under N+Fr and L+Fr treatments from their corresponding values under N and L treatments, respectively.

### Analysis of Differentially Expressed Proteins Related to Auxin and GAs

**FIGURE 7:**
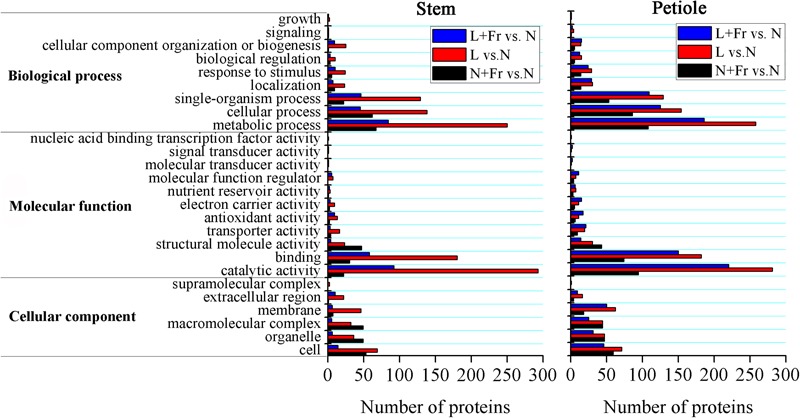
Differential protein expression analyses of soybean stem and petiole in different treatments. N, N+Fr, L, and L+Fr denote normal light (normal PAR and normal R/Fr ratio), normal light plus far-red light (normal PAR and low R/Fr ratio), low light (low PAR and normal R/Fr ratio), and low light plus far-red light (low PAR and low R/Fr ratio), respectively.

We employed TMT 10-plex labeling and LC-MS/MS to characterize the proteomic profiles of the soybean stems and petioles in different intensity and quality conditions. In total, 10,743 protein groups were identified, among which 9,349 proteins were quantified. A change of over 1.5-fold or a cutoff of less than 0.66-fold was considered statistically significant (**Supporting Information S1**). Differentially accumulated proteins were annotated and classified according to the biological process, molecular function, and cellular component (**Figure 7**). Among the quantified proteins, 101 proteins were up-regulated and 28 proteins were down-regulated in N+Fr vs. N in the stem; 316 proteins were up-regulated and 281 proteins were down-regulated in L vs. N in the stem; 105 proteins were up-regulated and 65 proteins were down-regulated in L+Fr vs. N in the stem; 161 proteins were up-regulated and 98 proteins were down-regulated in N+Fr vs. N in the petiole; 310 proteins were up-regulated and 306 proteins were down-regulated in L vs. N in the petiole; and 210 proteins were up-regulated and 257 proteins were down-regulated in L+Fr vs. N. This study examined the differentially expressed proteins associated with auxin and GAs that regulate the stems and petioles and found that the auxin-repressed superfamily protein and the GA-regulated protein were related to IAA and GAs. The auxin-repressed superfamily proteins in the stems and the petioles were significantly up-regulated under N+Fr, L, and L+Fr treatments with respect to their corresponding values under N treatment. By contrast, the GA-regulated protein, which appeared in the stems under L treatment, was significantly down-regulated. These results clarified the higher IAA level in the stems and petioles under N treatment than that under N+Fr, L, and L+Fr treatments and the higher GA1 level in the stem under N treatment than that under L treatment (Park and Han, 2003).

## Discussion

### Light Intensity or Quality: Which Was the Main Driver of the Changes in Soybean Morphologies under Shade Conditions?

Increasing the plant density and applying crop intercropping are effective strategies for improving the crop yield per unit land area in developing countries (Yang et al., 2015; Xie et al., 2017). However, these strategies are typically hindered by shade conditions (Li et al., 2014). Many studies have identified that shading promotes the stem and petiole growth while diminishing the leaf area (Gommers et al., 2013; Kurepin et al., 2015); however, few studies focused on the effects of uncoupling light intensity and quality from shading effects on the plant morphology. A reduced PAR irradiance coupled with either normal R/Fr or low R/Fr ratio significantly promoted seedling height elongation and decreased the *M*_A_ of soybean (**Figures 2B,C**). These results implied that light intensity played a vital role in regulating soybean seedling height and leaf weight per unit area. Previous studies also reported that the leaf morphology is significantly affected by reduced light intensity (Kurepin et al., 2007b). Although a reduced R/Fr ratio or a low PAR promotes stem elongation (Franklin and Whitelam, 2005), the effect of the light intensity on soybean height was greater than that of the light quality (R/Fr ratio) under shade conditions (**Figure 2B**).

A reduced R/Fr ratio coupled with a normal or low PAR promoted the petiole elongation of soybean, as previously showed in other plants (Ruberti et al., 2012; Gommers et al., 2013). The petiole length presented a nearly twofold increase compared with that when only the R/Fr ratio varied (under normal or low PAR). This finding suggested that a low R/Fr ratio was important for the canopy shade-induced petiole elongation of soybean.

Nevertheless, the stem, leaf, and petiole of soybean exhibited different responses to varying light intensities and qualities (R/Fr ratio), and these responses were likely regulated by endogenous hormones and molecular regulation networks (Vandenbussche et al., 2005; Sheerin and Hiltbrunner, 2017). However, previous studies have mainly focused on the effects of a reduced R/Fr ratio on hypocotyl or shoot elongation by analyzing the signal transduction pathway toward a phytochrome-mediated elongation in response to a reduced R/Fr ratio (Ruberti et al., 2012; Gommers et al., 2013).

### Light Intensity or Quality: Regulating the Matter Assimilation and Partition of Soybean

Photosynthesis, which is the source of biomass formation, depends on both light quantity and quality (Zhen and Van Iersel, 2017). The amount of biomass is the most direct measure of plant performance as a product of growth (Rodríguez-López et al., 2014); therefore, changes in the biomass can be an indicator of the plant response to the light availability under different light environments. In this study, a lower R/Fr ratio (increased far-red light) significantly increased the biomass under N+Fr or L+Fr treatments compared with their corresponding values under N or L treatments, respectively (**Figure 2E**). The reduced R/Fr ratio or increasing Fr in normal light or low light increased *M*_A_ (**Figure 2C**), which can indirectly promote biomass accumulation by increasing the radiation interception. This result was consistent with previous studies that far-red radiation promotes plant net assimilation of other species (Park and Runkle, 2017). These results were attributed to the high photosynthetic rate and quantum yield of PSII (**Figures 3, 4A** and **Table 1**). Zhen and Van Iersel (2017) also confirmed that far-red light can increase the photosynthetic efficiency of lettuce species. Similar to the Emerson enhancement effect, the photosynthetic efficiency of short wavelength (λ < 685 nm) can be improved by adding light with a long wavelength (λ > 700 nm) (Zhen and Van Iersel, 2017).

In addition, the lower R/Fr ratio under shade condition was similar to the increased far-red light (long wavelength) under low PAR environment. PSII (absorbing wavelengths between 400 and 680 nm and maximally at around 680 nm) and PSI (absorbing Fr light at above 700 nm) operate in series to facilitate photosynthesis in higher plants (Laisk et al., 2014). Given that PSII determines the rate of electron supply to PSI, an insufficient excitation of PSII with only Fr radiation strictly limits the overall quantum yield of photosynthesis (Hogewoning et al., 2012). Therefore, the added Fr under normal and low PAR conditions (N+Fr and L+Fr treatments) possibly contributed to the increased soybean net assimilation (compared with the case without added Fr under N and L treatments) by balancing the excitation between PSI and PSII.

**FIGURE 8:**
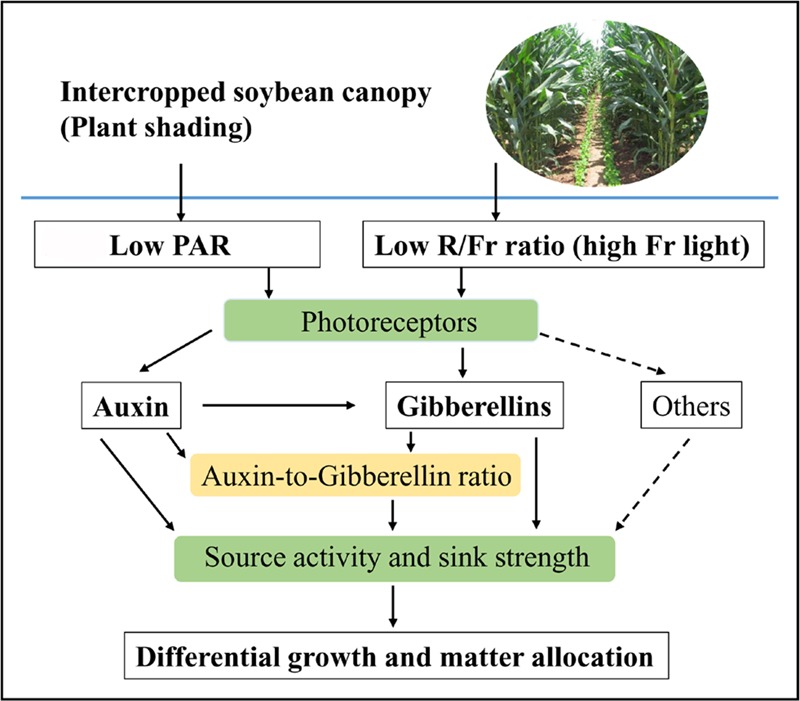
Schematic representation of auxin (IAA), gibberellins (GAs), and IAA-to-GA ratio as signals for light intensity and quality in regulating soybean growth and matter partitioning. Arrows of solid line represent the regulating directions of light intensity and quality on soybean growth and matter allocation in this paper. Arrows of dotted line represent that light intensity and quality may affect differential growth and matter allocation by other hormones.

Photosynthetic carbohydrate partitioning can reflect the response of each plant organ to different light environments. Plant shading generally leads to the allocation of carbohydrates toward stem or petiole elongation at the expense of the growth of the rest of the plant (Gommers et al., 2013). However, light intensity and quality exerted varied effects on the biomass allocation traits. Normal PAR and low PAR improved LMF and SMF, respectively. A reduced R/Fr ratio under either normal or low PAR can increase the PMF (**Figure 2F**). These results were consistent with the morphological trends of the stems, leaves, and petioles in response to different light environments (**Figure 2**). Furthermore, Rodríguez-López et al. (2014) posited that biomass accumulation increases linearly with increasing total light intensity, and that leaf morphological (including leaf biomass) changes are more responsive to light intensity than light quality. The response of the matter assimilation and partition to the changes of light intensity and quality may relate to the source activity and sink strength, which are regulated by auxin and GA metabolic networks (Smith, 2000; Yu et al., 2015; **Figure 8**).

### Light Intensity or Quality: Their Effects on the IAA and GA1 Levels of the Leaf, Stem, and Petiole of Soybean

Shading induces morphological changes, such as in auxin and GA hormone pathways (Ruberti et al., 2012). In this experiment, the endogenous IAA levels of soybean leaf were significantly increased under normal PAR with respect to their corresponding levels under low PAR; however, no significant differences were found for different R/Fr ratios under low PAR conditions (**Figure 5A**). This findings agreed with the results of previous studies (Kurepin et al., 2007b). In addition, light intensity and quality (R/Fr ratio) played equally important roles in regulating the GA1 levels of the soybean leaves (**Figure 5B**). Kurepin et al. (2007b) also reported that a low R/Fr ratio significantly increased the endogenous GA1 levels of *Helianthus* leaves. The data on leaf traits (**Figure 2C**) revealed that the change trends of the IAA level and IAA-to-GA1 ratio of the leaf were strongly similar to the trends of *M*_A_ of the leaf and LMF (**Figures 5A, 6A**). Thus, the *M*_A_ of the leaf and the biomass, which were mainly regulated by light intensity affecting IAA content and IAA-to-GA1 ratio, controlled plant cell division and elongation by regulating the expression of a vast number of genes (Sandalio et al., 2016).

A reduced R/Fr ratio and low PAR significantly decreased the IAA and GA1 levels in the top internode of the stem with respect to their corresponding levels under normal PAR (**Figures 5C,D**). However, several studies reported that reducing the R/Fr ratio from normal to low significantly increases the IAA and GA1 levels of sunflower hypocotyls or internodes under low and normal PAR irradiances (Kurepin et al., 2007a,b). These contradictory results may be due to the plant material and the treatment time. Analysis of differentially expressed proteins related to auxin and GAs revealed that the up-regulation of the auxin-repressed superfamily protein and the down-regulation of the GA-regulated protein affected the IAA and GA1 levels of the top internode under different treatments (**Table 3**). Park and Han (2003) also showed that the expression of auxin-repressed protein gene is negatively associated with hypocotyl elongation (Park and Han, 2003). In addition, the stem elongation and matter accumulation in different light environments may be influenced not only by levels of a specific hormone, but also by its interactions with other hormones (Kurepin et al., 2007b; Sandalio et al., 2016). Therefore, the change trends of the IAA-to-GA1 ratio were similar to the trends of stem height and SMF (**Figures 2B,F, 6B**) under N, L, and L+Fr treatments except N+Fr treatment. These results indicated that the stem elongation was regulated by IAA and/or GA interactions with other hormones besides the IAA-to-GA1 ratio in different light intensity and quality conditions (**Figure 8**). For example, brassinosteroid is considered as another factor stimulating elongation (Que et al., 2017).

**Table 3:** Differentially expressed proteins in the stems and petioles that were associated with auxin and GA regulation under different light intensity and quality treatments identified by TMT with LC-ESI-MS-MS/MS.

Accession	Description	Stem			Petiole		
		N+Fr vs. N	L vs. N	L+Fr vs. N	N+Fr vs. N	L vs. N	L+Fr vs. N
I1JWV1	Auxin-repressed superfamily protein	3.196	3.157	2.536	1.806	2.430	2.001
I1MEL3	PREDICTED: GA-regulated protein	–	0.399	–	–	–	–

*The horizontal line represents no significant level at more than 1.5-fold change or less than 0.66-fold cutoff of the differentially expressed proteins. N, N+Fr, L, and L+Fr denote normal light (normal PAR and normal R/Fr ratio), normal light plus far-red light (normal PAR and low R/Fr ratio), low light (low PAR and normal R/Fr ratio), and low light plus far-red light (low PAR and low R/Fr ratio), respectively.*

Limited information is available on the effects of light intensity and quality on IAA and GA1 levels, which regulate the petiole elongation. In this experiment, the change trends of the IAA levels of the soybean petiole under different treatments were consistent with those of the top internode of the stem (**Figures 5C,E**). This phenomenon may also be due to the up-regulation of the auxin-repressed superfamily protein under low PAR or R/Fr ratio (**Table 3**). A lower R/Fr ratio significantly increased the GA1 level of the petiole under normal PAR or low PAR conditions (**Figure 5F**). This phenomenon was similar to stem elongation, which is mediated by increased GA1 levels under a lower R/Fr ratio condition (Steindler et al., 1999). Although the changes in the GA1 level of the petiole were not consistent with the trends of the petiole length and PMF under different light environments, good opposite trends were observed among petiole length (**Figure 2D**), PMF (**Figure 2F**), and IAA-to-GA1 ratio (**Figure 6C**). This result also implied that hormonal crosstalk plays an important role in the effect of light environment on plant morphology (Vandenbussche et al., 2005). IAA can regulate concentration of GA1 in elongating internodes, and the removal of IAA reduces the concentration of GA1 (Ross et al., 2000; Wolbang and Ross, 2001). Therefore, the reduced R/Fr ratio promoted the growth of the petiole length by decreasing the IAA-to-GA1 ratio.

## Conclusion

Lower PAR and reduced R/Fr ratio were equally important for increasing soybean seedling height and SMF. However, the growth (*M*_A_ and LMF) of soybean leaves was greatly influenced by light intensity. Petiole elongation and PMF were mainly regulated by light quality (R/Fr ratio). Reduced R/Fr ratio (increased Fr light) improved the soybean biomass under normal or low PAR conditions by increasing the photosynthetic assimilation rate and quantum yield of PSII. The change trends of the IAA level and IAA-to-GA1 ratio of the leaf were strongly similar to the trends of *M*_A_ of the leaf and LMF. The trends of IAA and GA1 levels in the petiole were not consistent with the morphology traits and the biomass allocation under different light intensity and quality conditions. By contrast, the change trends of the morphology traits and the biomass allocation were strongly similar to the IAA-to-GA1 ratio in the petiole of soybean. In addition, the change trends of the IAA-to-GA1 ratio were similar to the trends of stem height and SMF under N, L, and L+Fr treatments except N+Fr treatment. This outcome may be due to other hormone interactions besides IAA-to-GA1 affecting stem elongation and matter accumulation. These results implied that the IAA-to-GA1 ratio was an important indicator of light intensity or light quality for regulating soybean growth and matter partition. Therefore, the dynamic trend of the IAA-to-GA1 ratio or its interactions with other hormones as a signal for regulating plant growth under different light intensity and quality conditions must be investigated in future works by using genomics, proteomics, and metabolomics.

## Author Contributions

FY, YF, XWu, YC, and QL performed the experiment. LF, JC, and ZW performed some experiments. FY, XWa, TY, WL, JL, JD, KS, and WY conceived the original research plans. FY and WY designed the experiments, analyzed the data, and wrote the article. All authors read and approved the final manuscript.

## Conflict of Interest Statement

The authors declare that the research was conducted in the absence of any commercial or financial relationships that could be construed as a potential conflict of interest.

## Supplementary Material

SUPPORTING INFORMATION S1 Differential protein expressed statistics of soybean stem and petiole in different treatments.
